# Intrinsic Negative Magnetoresistance and Broadband Photoresponse in Magnetic van der Waals Crystal TaFeTe_2_

**DOI:** 10.1002/advs.202523555

**Published:** 2026-01-27

**Authors:** Changcun Li, Jiazhen Wu, Sirong Lu, Lei Liang, Wenying Zhou, Bingke Zhang, Degang Zhao, Fucai Liu

**Affiliations:** ^1^ School of Materials Science and Engineering University of Jinan Jinan China; ^2^ Yangtze Delta Region Institute (Huzhou) and School of Optoelectronic Science and Engineering University of Electronic Science and Technology of China Chengdu China; ^3^ State Key Laboratory of Quantum Functional Materials Department of Materials Science and Engineering Southern University of Science and Technology Shenzhen China; ^4^ Guangdong Provincial Key Laboratory of Functional Oxide Materials and Devices Southern University of Science and Technology Shenzhen China; ^5^ Department of Physics Southern University of Science and Technology Shenzhen China

**Keywords:** broadband photodetections, intrinsic negative magnetoresistances, spin glasses, van der Waals materials

## Abstract

2D van der Waals (vdW) materials with broken symmetry, such as MM′Te_2_ (where M═Nb, Ta, and M′ = Fe, Co, Ni), have attracted considerable research interest due to their unique magnetic structures and optical‐phonon‐induced phase transitions, providing a versatile platform for discovering novel physical phenomena. Here, we report the synthesis of TaFeTe_2_ single crystals that can be mechanically exfoliated to the 2D limit. The as‐grown crystals display characteristic spin‐glass behavior and intrinsic unsaturated negative magnetoresistance up to 9 T, likely arising from the orbital effect associated with variable‐range hopping transport and/or magnetic disordering. Notably, TaFeTe_2_‐based photodetectors exhibit robust photocurrent responses in the sample's interior under self‐powered conditions, primarily arising from an additional charge separation mechanism induced by the optical‐phonon‐triggered non‐centrosymmetric phase. Under standard bias, the detectors achieve a high responsivity of 0.18 A W^−1^ and broadband photoresponse spanning the visible to mid‐infrared spectrum. These features highlight the material's strong potential for advanced multifunctional optoelectronic applications.

## Graphical abstract

2D van der Waals (vdW) materials MM′Te_2_ with broken symmetry attract great interest for unique magnetic structures and optical‐phonon‐induced phase transitions. We report mechanically exfoliable TaFeTe_2_ single crystals, exhibiting spin‐glass behavior, intrinsic unsaturated negative magnetoresistance up to 9 T, self‐powered internal photocurrent, and visible‐mid‐infrared broadband photoresponse, showing great potential for advanced multifunctional optoelectronics.

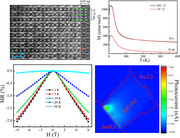

## 1 Introduction

Materials with broken symmetry in their electronic band structures demonstrate unique transport and photoelectric response phenomena. These materials hold great promise for enabling multifunctional next‐generation technologies, including macroscopic electrical, optical, magnetic, ferroelectric, and topological properties, etc [1, 2, 3]. The demand for miniaturization of electronic components has revealed the limitations of conventional materials, characterized by issues like short channel effects, high leakage current, and high contact resistance for high‐density logic devices [4, 5, 6, 7]. To address these challenges, there is a pressing need to explore novel nanomaterials that can mitigate these limitations in traditional electrodes, semiconductors, and dielectrics. 2D materials offer advantageous morphological and physicochemical properties owing to their atomically thin structures, exhibiting exotic physical phenomena such as the quantum Hall effect and quantum anomalous Hall effect. They provide a good material platform for studying the internal interactions between phonons, electrons, spins, and energy valleys [8, 9, 10, 11]. Moreover, 2D materials also possess high carrier mobility, dangling‐bond‐free surfaces, unusual optical features, and various band gaps, presenting great potential for the construction of high‐performance, broadband, and large‐scale integrated electronic and photoelectronic devices [5, 12, 13, 14]. Driven by these remarkable physical traits and extensive application prospects, an increasing number of novel 2D materials are being proposed and predicted for next‐generation device applications [15, 16, 17, 18].

Transition metal chalcogenides (TMCs), as one of the typical 2D materials, possess diverse chemical compositions and crystal structures [14, 19, 20]. Among them, group‐VB (V, Nb, and Ta) element‐based TMCs exhibit typical metallic behaviors and possess rich physical properties (superconductivity, charge density wave, magnetism, etc), which has aroused extensive interest in various fields [21, 22, 23, 24]. Ternary transition metal chalcogenides (TTMCs), which are produced by reducing metal TMCs with magnetic transition metals (Fe, Co, Ni, etc.), present rich compositions and magnetic structures, as well as controllable and variable electronic structures and symmetries, providing new avenues for discovering novel physical phenomena and designing new photodetectors with better performance [25, 26, 27, 28]. MM′Te_2_ as a representative layered TTMC, possesses an inversion center created by a mirror with two gliding planes and a double spin or helical axis, resulting in prohibited Weyl fermions with a small band gap [29, 30, 31]. The glide plane along the *c‐*axis and the associated rotational and helical axes will be completely lost after accounting for atomic displacements induced by optical phonons, which breaks the inversion symmetry, leading to a non‐centric space group of *Pm2a* with lower symmetry. These characteristics offer a great potential for the study of novel physical phenomena, as well as for overcoming the basic performance limitations of traditional photodetectors and developing high‐performance broadband photodetectors.

In this work, we successfully synthesize a new 2D vdW single crystal of TaFeTe_2_ using the chemical vapor transport (CVT) method and conduct a comprehensive investigation including crystal growth and characterization, electrical transport properties measurements, and photoelectronic properties analysis. The synthesized sample displays a typical spin‐glass behavior and an intrinsic unsaturated negative magnetoresistance (nMR) ascribed to the orbital effect associated with variable‐range hopping (VRH) transport and/or the magnetic disordering effect. The robust photocurrent (PC) responses are observed for the prepared TaFeTe_2_ photodetector in a self‐powered mode. Such a photoelectric phenomenon is interpreted as a result of an additional charge separation mechanism, probably due to optical‐phonon‐induced phase transition. Moreover, in the conventional mode, the TaFeTe_2_ photodetector also exhibits a fast response time, high responsivity, and broadband photo‐responses from the visible region to the mid‐infrared region.

## 2 Results and Discussion

**FIGURE 1:**
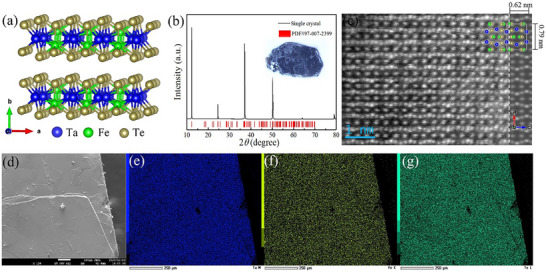
Structural and chemical information of TaFeTe_2_. (a) Crystal structure. (b) XRD pattern measured on a single‐crystalline sample (shown in the inset) with only the *a‐c* plane exposed to X‐rays. (c) The HAADF image of (010) plane for TaFeTe_2_ single crystal (The left panel shows the experimental image after sample drift correction, and the right panel shows the simulated image using the JMultiSlice software package). (d) SEM image and corresponding EDX elemental mappings for Ta (e), Fe (f), and Te (g).

Figure 1a presents the crystal structure of the as‐prepared TaFeTe_2_, in which the Ta‐Fe network is sandwiched by two interlaced Te layers, creating tetrahedral/octahedral coordination for Ta/Fe atoms. In the Ta‐Fe network, one Ta atom is bounded with four Fe atoms and one Fe atom is bounded with four Ta atoms and one Fe atom, resembling the structure of FeNbTe_2_ reported recently [32]. The vdW layers are stacked along the *b*‐axis with a large layer spacing of ∼1.7 Å. Figure 1b displays the X‐ray diffraction (XRD) pattern of an as‐prepared bulk TaFeTe_2_ single crystal (inset), demonstrating that the sample is of high quality. All diffraction peaks are indexed to the (*0l0*) crystallographic orientation. To confirm the microscopic lattice structure and chemical composition of the prepared sample, transmission electron microscopy (TEM) and energy‐dispersive X‐ray spectroscopy (EDX) analysis were performed. As shown in Figure 1c, clear and distinct atomic sites could be well identified in the (010) plane, and the alternating bright and dark sites are due to the different atomic numbers of Ta, Fe, and Te. The microscopic structural features of (100) and (001) planes are also examined in Figure S1, and vdW gaps can be clearly observed. The lattice parameters are measured as *a* = ∼7.9 Å, *b* = ∼7.3 Å, *c* = ∼6.2 Å, which are consistent with the XRD result and TaFeTe_2_ crystal structure [33]. Figure 1d demonstrates the scanning electron microscope (SEM) image of the bulk TaFeTe_2_ single crystal. The corresponding elemental mapping analyses of Ta (e), Fe (f), and Te (g) elements are also performed, demonstrating a uniform distribution of these elements.

**FIGURE 2:**
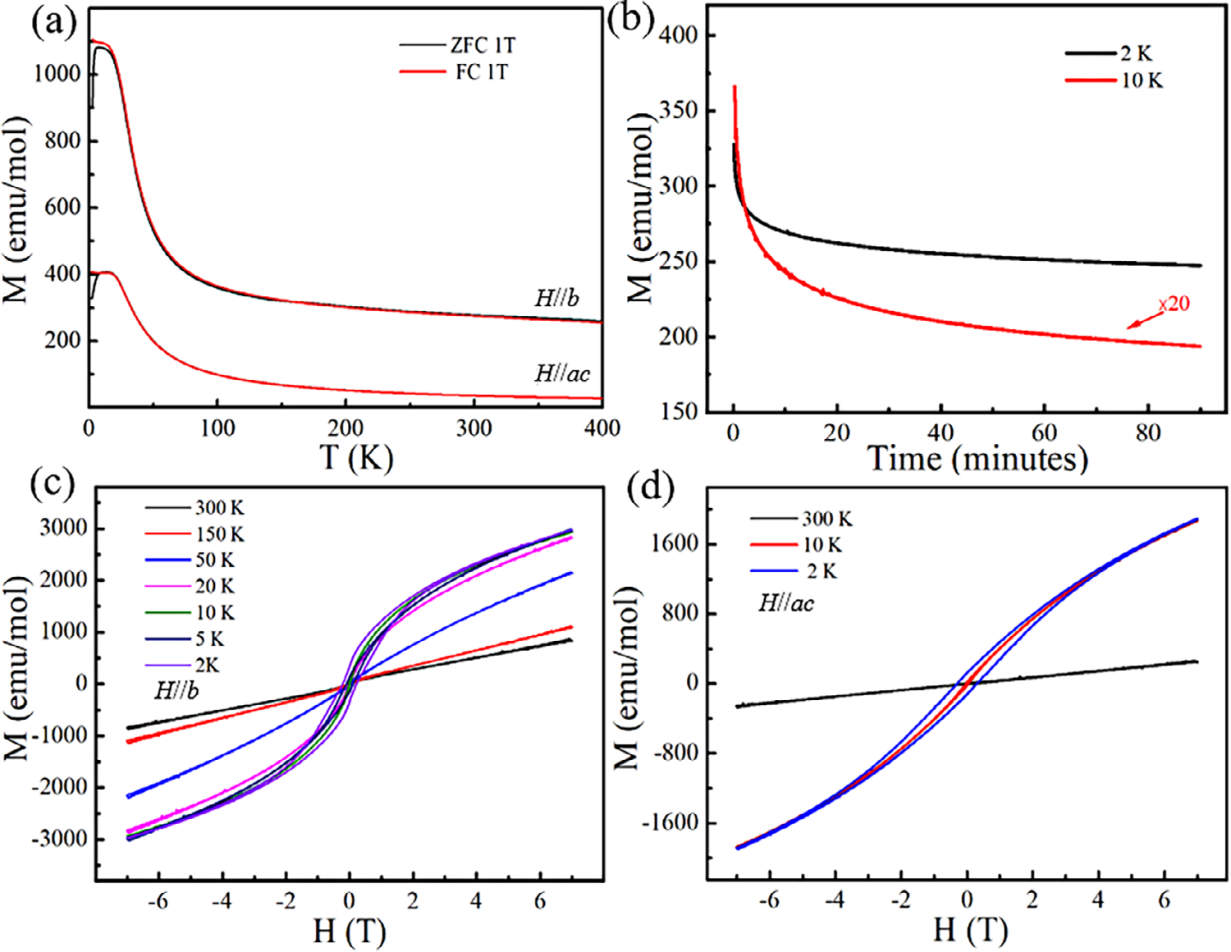
Magnetic properties of TaFeTe_2_. (a) Magnetization vs. temperature in a field of 1 T along *b*‐axis and *a–c* plane. (b) Thermoremanent magnetization curves at different temperatures after the samples were field‐cooled in a field of 7 T. The relaxations were measured immediately after the field was removed. Magnetic hysteresis loops at various temperatures with the field applied along (c) *b*‐axis and (d) *a–c* plane.

The magnetic transport behavior of TTMCs is closely related to their crystal structure and elemental coordination environment. Given this intrinsic correlation, TaFeTe_2_ crystals are anticipated to exhibit intriguing magnetic behaviors, as displayed in Figure 2. Figure 2a shows the temperature‐dependent magnetization of bulk TaFeTe_2_ under a field of 1 T applied parallel to the crystallographic *b*‐axis or *a‐c* plane. As the temperature decreases, the magnetization sharply rises and reaches a plateau at around 20 K. A clear splitting of the branches for zero field cooling (ZFC) and field cooling (FC) is observed at low temperatures, which is a typical feature of spin glass. To further confirm the spin glass behavior, time‐dependent residual magnetization after removing the external magnetic field at low temperatures was measured. As expected, TaFeTe_2_ exhibits a slow relaxation with a long relaxation time (Figure 2b). Figure 2c,d depicts the field dependences of magnetization for a TaFeTe_2_ single‐crystal sample along the *b*‐axis and *a‐c* plane, respectively. Both directions exhibit clear hysteresis loops, with a coercive field (*H*c) of ∼0.22 T along the *b*‐axis and ∼0.28 T within the *a‐c* plane at 2 K, respectively. As the temperature increases, *H*c gradually decreases. At a higher temperature of ∼ 50 K, the *H*c approaches zero, and the *M‐H* curve exhibits a linear correlation, showing a paramagnetic behavior.

**FIGURE 3:**
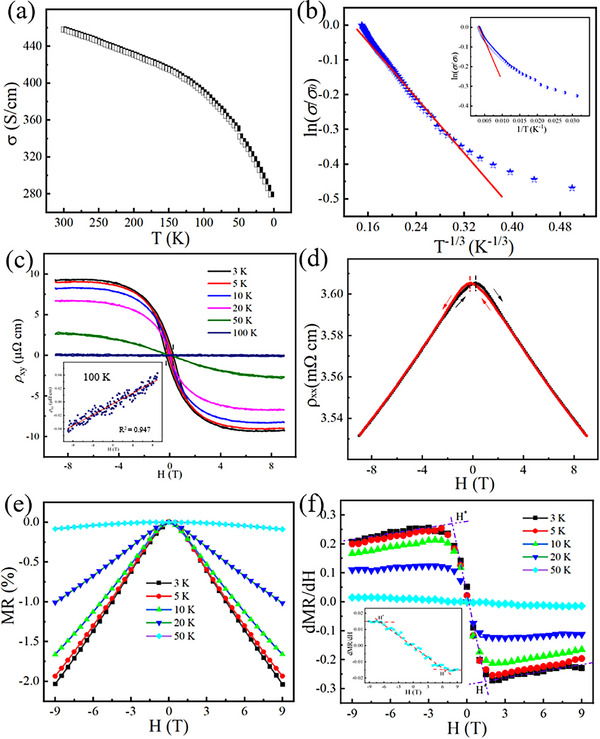
Transport performance of TaFeTe_2_. (a) Temperature dependence of the electrical conductivity and (b) Analysis of the temperature‐dependent electrical conductivity with the 2D VRH model (inset: the high‐temperature data fitted using the nearest‐neighbor hopping law, ln(*σ*)‐*T* ^−1^). (c) Field dependence of the Hall resistivity at various temperatures (inset: field dependence of the Hall resistivity at 100 K). (d) Field dependence of the longitudinal resistivity at 3 K. Field dependence of the (e) MR and (f) dMR/d*H* at various temperatures.

Considering the close correlation between the crystal structure and transport properties (both magnetic and electrical), after clarifying the magnetic transport characteristics of TaFeTe_2_, its electrical transport properties were systematically measured as shown in Figure 3. Figure 3a displays the temperature‐dependent electrical conductivity of a TaFeTe_2_ single crystal sample. As the temperature decreases from room temperature to 3 K, the electrical conductivity decreases slightly by ∼37%, which is different from conventional semiconductors. This peculiar electronic transport behavior, which is typically associated with intriguing physical phenomena, often appears in low‐dimensional disorder systems [32]. In localized systems, charge transport occurs through hopping processes. Theoretically, two types of hopping transport mechanisms are usually considered: the nearest‐neighbor hopping (NNH) and the variable‐range hopping (VRH). At high temperatures, the thermal activation of phonons exerts a significant influence on electrical conduction, and the electronic properties obey the NNH mechanism, which can be described by the following equation:

(1) $$\sigma_{T}=\sigma_{0}\exp\frac{-E_{a}}{k_{B}T}$$

where *E*_a_ is the typical energy difference between the adjacent localized states. The inset of Figure 3b displays the plot of normalized electrical conductivity *vs. T* ^−1^, which shows a linear relationship above ∼200 K. As the temperature decreases, deviations from the linear fitting occur. This indicates that a hopping process with a smaller energy spacing, such as VRH transport, becomes more dominant. According to the VRH theories, the temperature‐dependent conductivity can be expressed as [34, 35, 36]:

(2) $$\sigma \left(T\right)=\sigma_{0}exp{\left(-t/T_{0}\right)}^{1/\left(n+1\right)}$$

where *σ*_0_ is a constant, *n* depends on the density of states around the Fermi energy, and *T*_0_ is the characteristic temperature related to the energy barrier, and defined as [34]:

(3) $$T_{0}=13.8/\left(k_{B}N\left(E_{F}\right)\varepsilon^{2}\right)$$

where *N*(*E*_F_) stands for the density of states at the Fermi level, and ξ is the localization length in localized regimes [34]. As shown in Figure 3b, the logarithm of normalized electrical conductivity is linearly proportional to *T* ^−1/3^ in the mid‐temperature regime (∼30 K < T < ∼ 200 K), consistent with 2D Mott VRH theory. The value of *T*_0_ is estimated to be 27 K by fitting the experimental data using Equation (2). This value is three orders of magnitude lower than the reported *T*_0_ values in strongly localized regions, implying a weak carrier localization with a large localization length in the present sample [34, 37]. Upon further lowering the temperature (T < ∼ 30 K), a significant deviation and upwarp trend from the linear fitting scheme occurs, possibly due to the contribution of an additional hopping mechanism caused by magnetic interactions [32].

To gain comprehensive insights into the transport mechanisms of the TaFeTe_2_ single crystal, the field‐dependent Hall resistivity (*ρ*_xy_) at various temperatures was systematically measured, as shown in Figure 3c. At low temperatures, the Hall resistivity shows significant fluctuations at low magnetic fields. Interestingly, the Hall resistivity rapidly reaches an imperceptible peak as the magnetic field decreases from 0 to a negative magnetic field (or increases from 0 to a positive magnetic field), before gradually transitioning to a linearly field‐dependent regime beyond this peak at low temperature (<10 K). This characteristic behavior arises because the Hall resistivity of the magnetic crystalline material TaFeTe_2_ arises from two distinct contributions: the ordinary Hall effect (OHE) term stemming from the Lorentz force (external field) exerted by the external magnetic field on charge carriers, and the anomalous Hall effect (AHE) term, which is proportional to the magnetization. Therefore, *ρ*_xy_ can be described as [38]:

(4) $$\rho_{xy}\left(H\right)=R_{H}H+R_{s}M$$

where *R*_H_ denotes the Hall coefficient determined from a linear fitting of *ρ*_xy_ at high magnetic fields, and *R*_S_ represents the anomalous Hall coefficient of the magnetic component. To describe this phenomenon more intuitively, the *R*_H_ and *R*s values were extracted at 3K, which were determined to be 1.36 × 10^−11^ m^3^ C^−1^ and −4.83 × 10^−9^ m^3^ C^−1^, respectively. These results are consistent with the typical Hall transport behavior reported in magnetic materials [38, 39, 40]. Furthermore, the magnetic field corresponding to this peak exhibits a slight shift toward higher magnitudes with increasing temperature, leading to a monotonic evolution of the field‐dependent Hall resistance curve in the paramagnetic regime. Notably, the field‐dependent Hall resistance curve transitions to a distinct positive slope at 100 K (inset of Figure 3c). These observations support that the TaFeTe_2_ single crystal is likely to exhibit a multi‐carrier transport mechanism.

The hysteresis and VRH transport characteristics can also be reflected in the field‐dependent longitudinal resistance (*ρ*_xx_) as displayed in Figure 3d. As the magnetic field varies from −9 T to 9 T, the longitudinal resistivity increases to a maximum at *H*_c_ and then decreases rapidly. The longitudinal resistivity exhibits an identical change when the field direction is reversed, resulting in a bowtie shape for the field‐dependent longitudinal resistivity, consistent with the *M–H* hysteresis loop and Hall resistivity. The magnetoresistance (MR, defined as MR = [*ρ*(H)/*ρ*(0)‐1] × 100%) exhibits a monotonic change with temperature changing from 3 K to 50 K as shown in Figure 3e, presenting habitual nMRs without any sign of saturation. When the temperature exceeds 50 K, the variation in longitudinal resistance with the magnetic field becomes negligible (the amplitude is less than 0.1%). The nMR in TaFeTe_2_ reaches an extreme value of −2.0% at 3 K under a magnetic field of 9 T, which is an order of magnitude higher than that of NbFeTe_2_ with the same crystal structure [32]. The nMR of TaFeTe_2_ seems to show a ∼*H* ^2^ dependence in the weak magnetic field range and an ∼*H* dependence at high magnetic fields. To analyze this phenomenon more intuitively, the differentials of MR (dMR/dH) vs. magnetic field at various temperatures are presented in Figure 3f. In the weak magnetic field range (H < 1.5 T at 3 K), dMR/dH is proportional to the magnetic field, indicating a semi‐classical *H* ^2^ dependence for nMR. When the magnetic field exceeds the critical magnetic field (*H* ^*^), dMR/dH deviates from the semi‐classical behavior and saturates approximately to a constant value [41]. The nMRs observed in TaFeTe_2_ can be mainly attributed to both the orbital effect associated with VRH transport and the effect of spin glass. In the VRH transport regime, the carrier hopping paths are confined to a cigar‐shaped domain characterized by the hopping length R_h_ and width t = $\sqrt{R_{h}\varepsilon }$. The magnetic field‐induced resistance variation is dominated by the magnetic flux $\Phi_{M}=BR_{h}^{3/2}\varepsilon^{1/2}$ penetrating the cigar‐shaped region. This flux‐dependent modulation leads to the nMR exhibiting a quadratic dependence on the magnetic field in the weak‐field regime, and transforming into a linear dependence relationship in the high‐field regime [34]. In addition, the nMR arising from spin glass behavior can be expressed by the following expression [32, 42]:

(5) $$nMR=-k_{1}H\;\tanh\;\left(\frac{g\mu_{B}H}{2k_{B}T}\right)-k_{2}H^{2}$$

where *k*_1_ and *k*_2_ are both positive constants, *μ*_B_ is the Bohr magneton, *k*_B_ is the Boltzmann constant, and *g* is the Landé factor. $\tanh(\frac{g\mu_{B}H}{2k_{B}T})$ is proportional to *H* under low magnetic fields, and approaches the constant “1” under high magnetic fields. This field‐dependent evolution of the tangent term also gives rise to the transition of the nMR from a parabolic (quadratic) to a linear dependence on the magnetic field. The synergistic effect of the orbital effect (from VRH transport) and spin glass state endows TaFeTe_2_ single crystals with a higher nMR compared to similar materials [32]. As the temperature rises, the contributions of orbital effect and spin glass state to nMR gradually decreases, which in turn leads to a reduction in the absolute value of nMR with increasing temperature [25, 32, 42].

**FIGURE 4:**
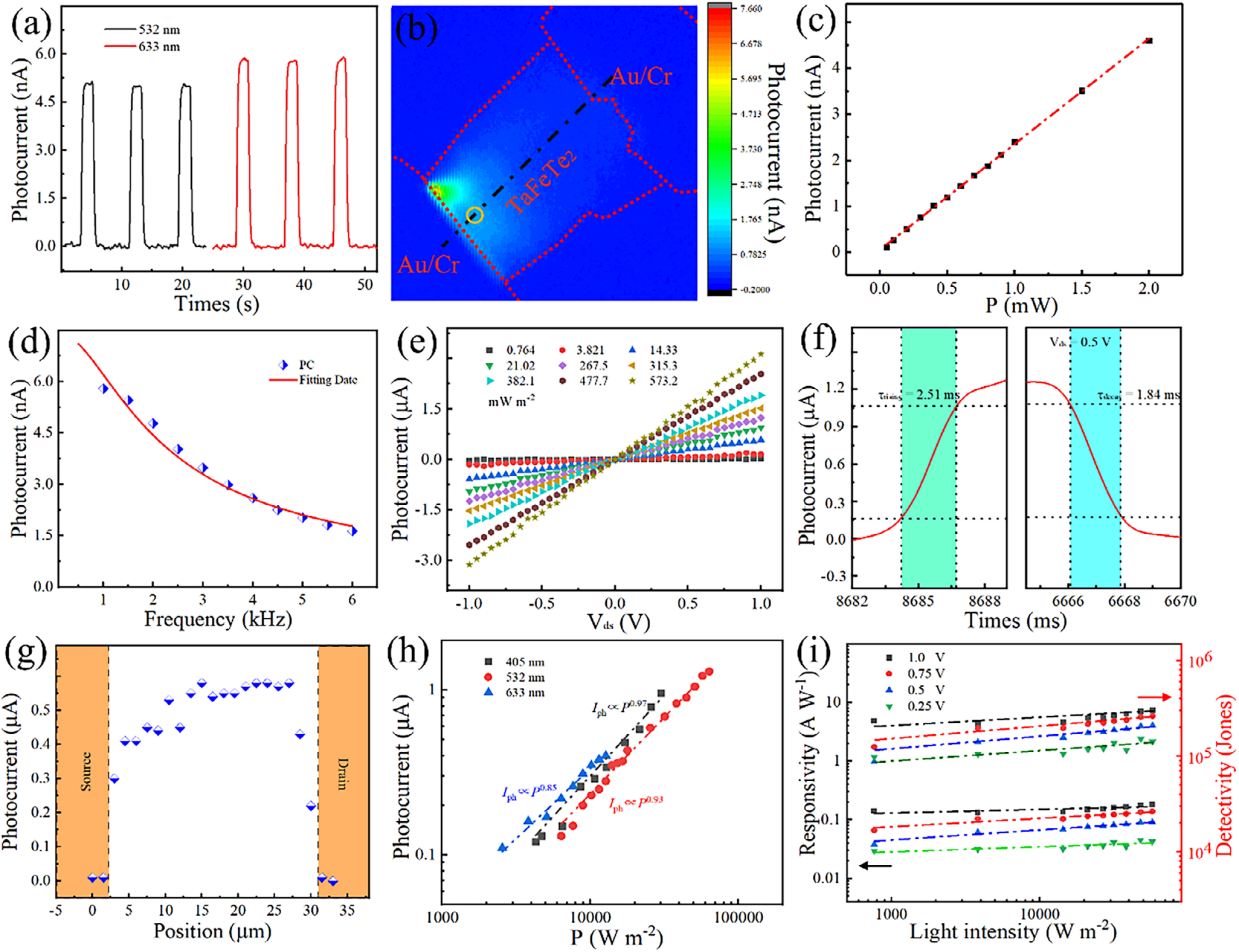
Optoelectronic properties of the prepared TaFeTe_2_ photodetector. (a) PC response under different wavelengths with 1 mW excitation power. (b) SPCM image under a 532 nm laser with 500 µW excitation power. (c) The PC response as a function of excitation power at the position masked as a golden circle in b. (d) Chopping frequency‐dependent PC response measurement with a lock‐in amplifier under 532 nm excitation. (e) *I*_ph_‐*V*_ds_ curves with various powers under 532 nm laser. (f) A single response circle of the photodetector is used to estimate the rising and decay times. (g) Position‐related photocurrent profile through the entire channel under 532 nm laser with a bias voltage of 1 V. (h) PCs vs. incident power under different laser wavelengths. (i) Laser intensity dependence of the responsivity and detectivity under different bias voltages deduced from the *I*_ph_‐*V*_ds_ curves in (e).

In addition to the above‐mentioned interesting transport properties, the TaFeTe_2_ crystal is highly anticipated for achieving broadband spectral responses and developing novel optoelectronic phenomena due to its exceptional attributes, including ultra‐narrow band gap (∼0.06 eV) and optical‐phonon phase transition characteristics [29, 30, 31]. Several‐layered TaFeTe_2_ flakes are obtained on SiO_2_/Si substrates by the mechanical exfoliation technique and used to fabricate photodetectors (Figure S2). The *I*_ds_‐*V*_ds_ curve of the prepared TaFeTe_2_ photodetector exhibits perfect linearity, suggesting a good ohmic contact between the channel material and electrodes. This indicates a good conductivity and efficient charge transport within the photodetector. The prepared TaFeTe_2_ photodetector presents a stable short‐circuit PC response (Figure 4a). To further investigate the origin of this response, a scanning photocurrent measurement (SPCM) pattern of the TaFeTe_2_ photodetector is recorded (Figure 4b) in an ambient condition with 532 nm excitation. The SPCM pattern reveals a distribution of PC response across the device, including the interfaces between the TaFeTe_2_ flake and metal electrode, as well as the inner regions of TaFeTe_2_ away from the metal electrodes. The extremum positive PC response is generated at one side of the metal‐TaFeTe_2_ junction, and the PC responses gradually decrease as the excitation moves away from the junction regions. This decrease is attributed to the weakening of the major charge separation mechanisms (e.g., built‐in field). These observations suggest that the photo‐thermoelectric (PTE) effect or/and photovoltaic (PV) effect play a crucial role in the short‐circuit PC generation [43, 44]. Notably, the robust positive PCs are observed when the excitation is irradiated on the inner regions of the prepared photodetector, far away from the metal‐TaFeTe_2_ junctions. The minimum PCs are obtained when the excitation is close to the other side of the TaFeTe_2_‐metal junction region. These results indicate the presence of an additional charge separation mechanism throughout the SPCM measurement. The non‐centric space‐group symmetry structure of TaFeTe_2,_ caused by the optical‐phonon effect, generates a robust spontaneous polarization field [29]. The interaction between the polarization field caused by the pyroelectric effect and the built‐in field caused by PTE or/and PV effect leads to the fascinating PC distribution observed in the entire channel of the TaFeTe_2_ photodetector. To visualize this phenomenon more intuitively, the position‐related PC responses along the line cut (masked as black in Figure 4b) are presented (Figure S3b). The responsivity and detectivity are calculated to be 71 µA W^−1^ and 5.8 × 10^6^ Jones, respectively, highlighting its significant potential for application in self‐powered photodetectors. Additionally, the power‐dependent PC response shows a good linear correlation in Figure 4c. The photodetector also exhibits a low response time of ∼0.12 ms, as measured by the standard chopping frequency‐dependent PCs (Figure 4d).

Figure 4e presents the bias voltage‐resolved PC response (defined as *I*_ph_ = *I*_light_—*I*_dark_, in which *I*_light_ and *I*_dark_ are the currents under illumination and dark state, respectively) of the prepared TaFeTe_2_ photodetector under illumination of 532 nm with different laser powers. The *I*_ph_ of the TaFeTe_2_ photodetector gradually increases with increasing bias voltage or laser power, demonstrating good stability. A time‐resolved *I*_ph_ curve over one cycle is presented in Figure 4f, showing a fast response with a rising time of 2.51 ms and a decaying time of 1.84 ms. Figure 4 g shows the position‐related *I*_ph_ across the entire channel under a bias voltage of 0.5 V. The *I*_ph_ originates from the entire channel, which is consistent with the photoconductive mechanism. Figure 4 h shows the relationship between the *I*_ph_ and incident laser intensity (*P*), where the *I*_ph_ is proportional to *P* ^θ^ and the fitted values of *θ* are close to 1 (derived from the power‐law relationship *I*_ph_∝*P* ^θ^), suggesting that the main source of *I*_ph_ is photo‐generated carriers generated by photon absorption of TaFeTe_2_. Meanwhile, the ideal θ value also indicates that the prepared TaFeTe_2_ possesses high crystal quality. To better evaluate the performance of the TaFeTe_2_ photodetector, the responsivity (*R*) and detectivity (D*) are calculated using the following equations:

(6) $$R=I_{\mathrm{ph}}/\left(P\times \mathrm{Adevice}\right)$$

(7) $$D^{*}=R\times \;\mathrm{Adevice}\;1/2\;\left(2\mathrm{qIdark}\right)1/2$$

where *P* is the laser power density, and *q* is the electronic charge. Figure 4i presents the light power density dependence of the responsivity and detectivity under different bias voltages. The highest responsivity, estimated to be ∼ 0.18 A W^−1^ under a bias voltage of 1 V, is comparable to the photodetectors based on other 2D semi‐metals and narrow band gap semiconductors [44, 45, 46, 47]. The prepared TaFeTe_2_ photodetector also shows great potential in broadband photodetection, as displayed in Figure S3. Due to the high *I*_dark_ in the prepared TaFeTe_2_ photodetector, its performance in terms of stability and low‐light detection is far away satisfactory. The construction of photodiodes consisting of p‐n, p‐i‐n, or Schottky junctions could easily achieve low *I*_dark_ and excellent optoelectronic properties through the formation of a junction barrier and built‐in electric field [5, 48].

## 3 Conclusions

In summary, we have successfully synthesized an exfoliable TaFeTe_2_ bulk single crystal and prepared photodetectors based on the flakes obtained via the mechanical exfoliation technique. The as‐prepared samples exhibit typical spin glass behavior and intrinsic unsaturated nMRs up to 9 T, which can be synergistically attributed to the VRH orbital effect and spin glass effect. The non‐centrosymmetric phase induced by the optical phonon may create an extra mechanism for charge separation, yielding robust PC responses in the inner regions of the TaFeTe_2_ photodetector under the self‐powered mode. Moreover, the TaFeTe_2_ photodetector under the conventional mode also demonstrates a high responsivity of 0.18 A W^−1^, and broadband spectral response up to 10.6 µm. These findings highlight the MM′Te_2_ category as an ideal platform for exploration of unique transport and photoelectric response phenomena, as well as for designing high‐performance broadband photodetectors.

## 4 Experimental Section

### 4.1 Sample Synthesis

High‐quality TaFeTe_2_ single crystals were synthesized using the CVT method. Specifically, stoichiometric amounts of high‐purity Fe (99.99%), Ta (99.99%) and Te (99.99%) powders were thoroughly ground and mixed in an agate mortar, and then sealed in an evacuated quartz tube (*Φ *1.3*15 cm) at a pressure of less than 10^−2^ Pa. Iodine (*I*_2,_ 99.9%) was added as the transport agent with a dosage of 5 mg per gram of the mixed starting materials to facilitate vapor‐phase mass transport. Then, the sealed quartz tube was placed in a two‐zone horizontal furnace, where a temperature gradient of 100°C was established between the source zone (750°C) and growth zone (650°C). The temperature ramp rate of the source zone was set to 5°C min^−1^ to avoid thermal shock to the quartz tube, and the system was held at the target temperature for 7 days to ensure the complete reaction and crystal growth. After the reaction, the quartz tube was rapidly quenched down to room temperature in water to preserve the crystal structure and prevent phase transformation. After that, the block‐like TaFeTe_2_ single crystals with metallic luster were collected from the growth zone of the quartz tube, and screened by using an X‐ray diffractometer (Bruker D8 Advance, Germany). Scanning electron microscope images and energy‐dispersive X‐ray analysis mapping images of the synthesized single crystals were obtained using an S‐4800 electron microscope (Hitachi, Japan). Transmission electron microscopy images were acquired by using a JEM‐ARM200F electron microscope. The morphology of thin flakes obtained by the mechanical exfoliation technique was investigated by optical microscopy and atomic force microscopy.

### 4.2 Physical Properties Measurement

Magnetizations were measured using a Magnetic Property Measurement System (MPMS, SQUID VSM Quantum Design). Electrical transport properties were measured using a Physical Properties Measurement System (PPMS, Quantum Design). The longitudinal resistances (*ρ*_xx_) and Hall resistances (*ρ*_xy_) were measured in the natural ribbons of TaFeTe_2_ single crystals using a standard four‐probe technique.

### 4.3 Device Fabrication and Measurements

The photodetectors were prepared by depositing Cr/Au (8/40 nm) electrodes onto the surface of exfoliated TaFeTe_2_ thin flakes using an electron beam evaporator. To guarantee the reliability and repeatability of the device performance, TaFeTe_2_ thin flakes underwent rigorous pre‐screening. First, the lateral dimension of the thin flakes should be greater than 60 µm to meet the processing requirements of the detector. Second, the selected thin flakes should possess a complete surface morphology (free of cracks, wrinkles, or other structural defects), as such imperfections could compromise the detector's ability to deliver its intrinsic performance. In addition, the thickness of the screened thin flakes was precisely controlled within the range of 60–80 nm, which is optimal for achieving broadband light absorption [44, 49]. Short‐circuit PC signals were detected using a lock‐in amplifier, and the laser beam was modulated with a mechanical chopper (233 Hz). The laser beam was focused by a 50× transmissive objective lens. Standard scanning PC measurements were performed under ambient conditions using a 532 nm laser with ∼ 3 µm spatial resolution. To measure the response time (*τ*) of the short‐circuit photocurrent, different chopping frequencies were used using an optical chopper. The PC response (*R_f_*) taken from a lock‐in amplifier as a function of the different chopping frequencies (*f*) was fitted with *R_f_* = *R*_0_/[1 + (2*πfτ*)^2^]^1/2^ to obtain the response time. Photoelectric characterizations of the prepared photodetectors under the conventional mode were performed by the semiconductor analyzer (FS‐Pro 380) under ambient conditions.

## Conflicts of Interest

The authors declare no conflict of interest.

## Supporting information




**Supporting File**: advs73873‐sup‐0001‐SuppMat.docx.

## Data Availability

The data that support the findings of this study are available from the corresponding author upon reasonable request.
