# Supplementary material for: Intrinsic Negative Magnetoresistance and Broadband Photoresponse in Magnetic van der Waals Crystal TaFeTe2
Source: Adv Sci (Weinh). 2026 Jan 27;13(18):e23555. doi: 10.1002/advs.202523555 (PMC13042978; doi:10.1002/advs.202523555)
Supplement: Supplementary file 1 — Supporting File: advs73873‐sup‐0001‐SuppMat.docx. [file ADVS-13-e23555-s001.docx]

# Intrinsic Negative Magnetoresistance and Broadband Photoresponse in Magnetic van der Waals Crystal TaFeTe_2_

Changcun Li, Jiazhen Wu*, Sirong Lu, Lei Liang, Wenying Zhou, Bingke Zhang, Degang Zhao*, and Fucai Liu*

Dr. C. Li, Dr. W. Zhou, Dr. B. Zhang, Prof. D. Zhao

School of Materials Science and Engineering, University of Jinan, Jinan, 250024, China

Dr. C. Li, L. Liang, Prof. F. Liu

Yangtze Delta Region Institute (Huzhou), and School of Optoelectronic Science and Engineering, University of Electronic Science and Technology of China, Chengdu, 611731, China

Dr. J. Wu

State Key Laboratory of Quantum Functional Materials, Department of Materials Science and Engineering, Southern University of Science and Technology, Shenzhen, 518055, China

Dr. J. Wu

Guangdong Provincial Key Laboratory of Functional Oxide Materials and Devices, Southern University of Science and Technology, Shenzhen, 518055 China

Dr. S. Lu

Department of Physics, Southern University of Science and Technology, Shenzhen 518055, China

*E-mail: wujz@sustech.edu.cn, mse_zhaodg@ujn.edu.cn, fucailiu@uestc.edu.cn

**Data analysis methods for electrical transport measurements**

Magnetoresistance (*MR*) is defined as (*ρ*_xx_ (*H*, *T*)- *ρ*_xx_ (*0*, *T*))/ *ρ*_xx_ (*0*, *T*), where *ρ*_xx_ (*H*, *T*) represents the resistivity in a magnetic field *H* under temperature *T*, and *ρ*_xx_ (*0*, *T*) is the resistivity under *T* without magnetic field. The measured field-dependent longitudinal resistances (*ρ*_xx, measure_) inevitably contains the contributions from both the MR effect and Hall effect, and similarly the measured field-dependent Hall resistances (*ρ*_xy, measure_) contains the contributions from both Hall effect and MR effect due to the not perfectly aligned electrodes for electrical transport properties. That is *ρ*_xx, measure_ = *ρ*_xx, L_ + *ρ*_xy, L_, and *ρ*_xy, measure =_ *ρ*_xy, H_ + *ρ*_xx, H_, where *ρ*_xx, L_, and *ρ*_xy, L_, represent the longitudinal resistivity contributed from MR effect and Hall effect respectively, and *ρ*_xy, H_, and *ρ*_xx, H_ are the Hall resistivity contributed from Hall effect and MR effect respectively. We analyzed the data on two equations: 𝜌_xy_ (*H*) = - 𝜌_xy_ (-*H*), and 𝜌_xx_ (*H*) = 𝜌_xx_ (-*H*), to separate the two contributions. The pure Hall resistivity was achieved by 𝜌_𝑥𝑦_ (𝐻) = (𝜌_𝑥𝑦, measure_(𝐻) – 𝜌_𝑥𝑦, measure_(-𝐻))/2, and the pure longitudinal resistivity was achieved by 𝜌_𝑥𝑥_ (𝐻) = (𝜌_𝑥𝑥, measure_(𝐻) + 𝜌_𝑥x, measure_(-𝐻))/2. To analyze the data in such a way, we measured both the 𝜌_𝑥𝑦, measure_ and 𝜌_𝑥𝑥, measure_ with a magnetic field swept from a negative field to a positive field and then from a positive field to a negative field.


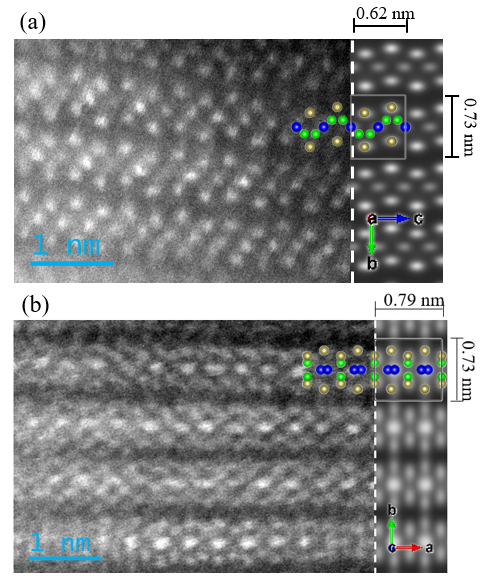


**Figure S1** The high-angle annular dark-field (HAADF) images of (a) (100) and (b) (001) planes for TaFeTe_2_ (The left panels show the experimental image and the right panels show the simulated image using JMultiSlice software package).


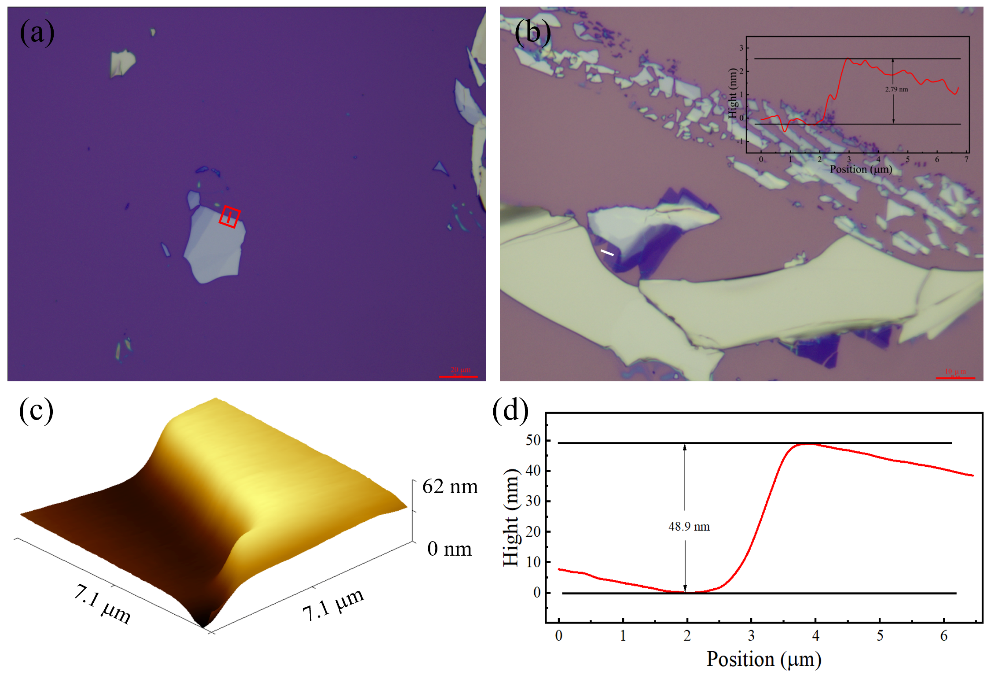


**Figure S2 Optical images and atomic force microscopy image of the exfoliated TaFeTe_2_ flakes on the SiO_2_/Si substrate.** (a, b) Optical images of the exfoliated TaFeTe_2_ flakes, (the inset of b is the height profile along the white line). (c) AFM image of the thin flake and (d) the obtained height profile. The scanning range is shown in (a) as a red box and red line, respectively.


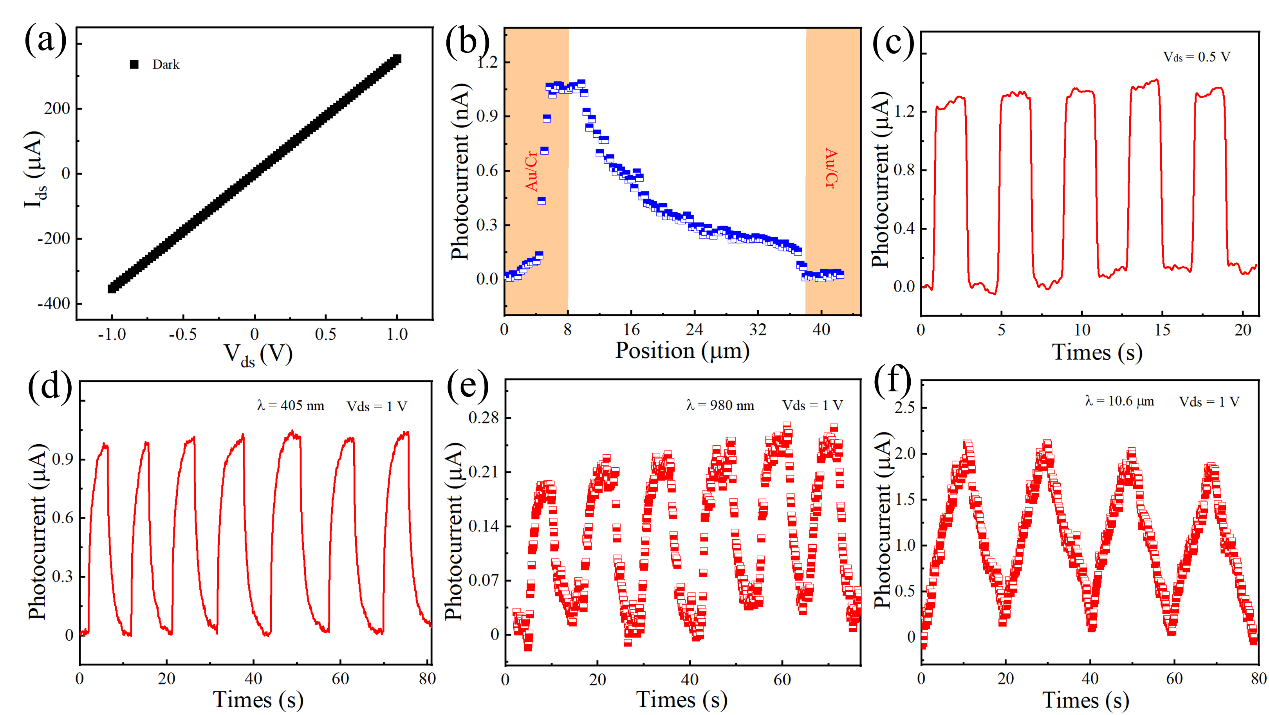


**Figure S3 Optoelectronic properties of the prepared TaFeTe_2_ photodetector.** (a) The measured *I*_ds_-*V*_ds_ curve in the dark state. (b) Photocurrent response of the TaFeTe_2_ photodetector along the line cut marked as black line in Figure 4b. Photoresponse of the photodetector for different excitation wavelengths (c: 532 nm@1.28 W cm^-2^; d: 405 nm@0.13 W cm^-2^; e: 980 nm@0.09 W cm^-2^; f: 10.6 μm@0.23 W cm^-2^) under a bias voltage of 0.5 V at room temperature.

Different tendencies in time-resolved photocurrent at different wavelengths are mainly attributed to the different instantaneous excitation energies. ^[1, 2]^ In the infrared region (980 nm), the ideal current plateau doesn’t appear but continues to climb with a lower speed as the laser is illuminated on the surface of the prepared detector, which is mainly owing to the obvious thermal effect of infrared light radiation. The appearance of non-equilibrium carriers owing to the activation of hot carriers inside the channel materials under continuous irradiation increases the photocurrent output of the prepared photodetector. Correspondingly, the current of the prepared photodetector also exhibits a slow decreasing process after the laser is turned off, which is also attributed to the slow reaction and gradual quenching of thermal carriers. As the further increase in wavelength (up to 10.6 μm), the excitation energy is lower that the bandgap of the channel material. The dominant mechanism for photocurrent generation is changed from photocurrent effect to photo-bolometric effect, which also results in a significant change in the time-resolved photocurrent curve.

**References:**

[1] J. Lai, Y. Liu, J. Ma, X. Zhuo, Y. Peng, W. Lu, Z. Liu, J. Chen, D. Sun, *ACS Nano* **2018**, 12, 4055.

[2] C. Li, J. Wu, R. Bian, G, Cao, E. Pan, Z. Liu, J. Yu, H. Hosono, F. Liu, *Adv. Funct. Mater.*, **2022**, 32, 2208531.
